# The Effect of Periodontitis on Dementia and Cognitive Impairment: A Meta-Analysis

**DOI:** 10.3390/ijerph18136823

**Published:** 2021-06-25

**Authors:** Haiying Guo, Shuli Chang, Xiaoqin Pi, Fang Hua, Han Jiang, Chang Liu, Minquan Du

**Affiliations:** 1 The State Key Laboratory Breeding Base of Basic Science of Stomatology (Hubei-MOST) & Key Laboratory of Oral Biomedicine Ministry of Education, School & Hospital of Stomatology, Wuhan University, Wuhan 430079, China; hayingguo@whu.edu.cn (H.G.); changshuli76@whu.edu.cn (S.C.); pixiaoqin@whu.edu.cn (X.P.); huafang@whu.edu.cn (F.H.); jianghan@whu.edu.cn (H.J.); 2 Center for Evidence-Based Stomatology, School & Hospital of Stomatology, Wuhan University, Wuhan 430079, China

**Keywords:** periodontal disease, periodontitis, dementia, cognitive impairment, meta-analysis

## Abstract

The association between periodontal disease and dementia/cognitive impairment continues to receive increasing attention. However, whether periodontal disease is a risk factor for dementia/cognitive impairment is still uncertain. This meta-analysis was conducted to comprehensively analyze the effect of periodontitis on dementia and cognitive impairment, and to assess the periodontal status of dementia patients at the same time. A literature search was undertaken on 19 October 2020 using PubMed, Web of Science, and Embase with different search terms. Two evaluators screened studies according to inclusion and exclusion criteria, and a third evaluator was involved if there were disagreements; this process was the same as that used for data extraction. Included studies were assessed with the Newcastle-Ottawa Scale (NOS), and results were analyzed using software Review Manager 5.2. Twenty observational studies were included. In the comparison between periodontitis and cognitive impairment, the odds ratio (OR) was 1.77 (95% confidence interval (CI), 1.31–2.38), which indicated that there was a strong relationship between periodontitis and cognitive impairment. There was no statistical significance in the effect of periodontitis on dementia (OR = 1.59; 95%CI, 0.92–2.76). The subgroup analysis revealed that moderate or severe periodontitis was significantly associated with dementia (OR = 2.13; 95%CI, 1.25–3.64). The mean difference (MD) of the community periodontal index (CPI) and clinical attachment level (CAL) was 0.25 (95%CI, 0.09–0.40) and 1.22 (95%CI, 0.61–1.83), respectively. In this meta-analysis, there was an association between periodontitis and cognitive impairment, and moderate or severe periodontitis was a risk factor for dementia. Additionally, the deterioration of periodontal status was observed among dementia patients.

## 1. Introduction

Dementia is characterized by a progressive decline in cognitive ability and interference with patients’ daily performance over time [1]. Cognitive impairment is a transitional condition between the expected cognitive decline of normal aging and dementia, and is highly likely to develop into dementia. The most common type of dementia is Alzheimer’s disease (AD), which accounts for 60–80% of all cases [2]. As a global health challenge, 82 million people will be diagnosed with dementia by 2030, and the global financial cost of dementia will rise to $2 trillion [3], which is a heavy burden on society and families.

Although the mechanisms of dementia are still unclear, increasing evidence indicates that inflammation plays a major role in dementia/cognitive impairment [4,5]. For example, a rise of IL-6, IL-1, TNF-α, and C-reactive protein has been observed in dementia patients [6,7]. Moreover, studies have shown that non-steroidal anti-inflammatory drugs (NSAIDs) can reduce the risk of AD [8]. In addition, microbes might also contribute to dementia and cognitive impairment [9,10,11]. For instance, *Chlamydia pneumoniae* [9] and *Borrelia burgdorferi* [10] have been found in the blood and cerebrospinal fluid of AD patients. Additionally, a case-control study showed that patients with infections were 2 times as likely to suffer from dementia as persons without infections [12].

Periodontal disease is not only a common chronic infectious and inflammatory oral disease, but also contributes to systemic diseases [13]. Nowadays, more and more attention has been paid to the association between periodontal disease and dementia/cognitive impairment. For example, participants with periodontitis were found to have a higher risk of dementia (hazard ratio (HR) = 1.16; 95% confidence interval (CI) = 1.01–1.32, *P* = 0.03) than those without periodontitis, even after adjustment for confounding factors [14]. Meanwhile, multivariate analyses showed that there was a negative correlation between cognitive scores and the proportion of periodontitis sites [15]. However, a cross-sectional study found that periodontal disease, even with deep lesion/severe bleeding, was not associated with cognitive test outcomes [16]. Overall, the sample size, study design, assessment of periodontal disease and dementia/cognitive impairment, and additional differences in controlled factors might contribute to this discrepancy.

Considering the incurability of dementia and the available interventions for periodontal disease, it is important to clarify whether periodontal disease is a risk factor for dementia. Therefore, we performed this meta-analysis to illustrate the effect of periodontitis on dementia and cognitive impairment, and to assess the periodontal status of dementia patients at the same time.

## 2. Materials and Methods

### 2.1. Search Strategy

A literature search was undertaken from the date of establishment of the database to October 19, 2020 using the following databases: PubMed, Web of Science, and Embase. Search terms in PubMed included the following key words: #1-(“periodontal disease” OR “periodontitis” OR “gingivitis” OR “oral health” OR “oral hygiene”); #2-(“dementia” OR “Alzheimer’s disease” OR “cognitive decline” OR “cognitive impairment”). Various search strategies in other specific databases are shown in the Supplementary Search Strategies S1. In addition, references of included articles and articles from conference abstracts were also manually retrieved to maximize the number of included studies. No gray literature was searched in our study. If there was a lack of relevant data in articles, we contacted the authors to acquire missing data.

### 2.2. Inclusion and Exclusion Criteria

Articles were included if they featured: (1) observational studies on the association between periodontal disease and dementia or cognitive impairment, including case-control, cross-sectional, and cohort studies; (2) periodontal disease measured by at least one of the following indexes: pocket probing depth (PPD), clinical attachment level (CAL), radiographic alveolar bone loss (RABL), marginal alveolar bone loss (MABL), the gingival index (GI), plaque index (PI), community periodontal index (CPI), bleeding on probing (BOP) and the gingival bleeding index (GBI); (3) dementia and cognitive function reported by at least one measure for cognitive function, such as the Mini-Mental State Examination (MMSE), Montreal Cognitive Assessment (MoCA), or Clock test and so on.

Exclusion criteria was as follows: (1) studies with missing data which did not mention the author or the author was contacted with no reply; (2) case reports, meeting abstracts and reviews; (3) studies reporting an overlap in the sample of participants; (4) studies not written in English.

### 2.3. Study Screening and Data Extraction

Two evaluators independently extracted data and screened the titles, abstracts, and full texts according to the inclusion and exclusion criteria. Disagreements were resolved by discussion or in consensus meetings with a third reviewer.

Extracted data included the name of the author and the time of publication, study design, characteristics of the population, indexes of periodontal disease and dementia, etc. Authors were contacted to obtain relevant information. In cases of multiple group studies, only the data related to periodontal disease and dementia were extracted.

### 2.4. Quality Assessment

The Newcastle–Ottawa Scale (NOS) was used to assess the included studies [17]. Three themes included eight items, with a score range of 0–9 points. Overall, studies with NOS scores of 1–3, 4–6, and 7–9 were judged to be of low, moderate, and high quality, respectively [18].

To obtain an intuitive profile of the included studies, we added the items of the NOS to the software Review Manager 5.2 (Cochrane Collaboration, Oxford, UK). If there was no score in an item, then the risk of the item was assessed as “unclear”. Otherwise, “low risk” was assessed.

### 2.5. Reporting Bias

Reporting bias was also assessed in this meta-analysis. If there were more than 10 studies in each meta-analysis, a funnel plot was used to assess publication bias. If the funnel plot was symmetric, there was no publication bias. However, if the funnel plot was not symmetric, potential reasons for this occurrence were analyzed further.

### 2.6. Data Pre-Processing

According to the similar definition of the CPI and the community periodontal index for treatment needs (CPITN), we used the CPI instead of the CPITN. Besides, we found PP was short for periodontal pocket, and loss of attachment (LoA) meant the same as CAL, so we used PPD and CAL instead of PP and LoA, respectively.

As shown in articles, the definition of periodontitis was various. In order to combine effect size reasonably, ≥1 tooth with periodontal pocket depth ≥ 4 mm or CAL ≥ 1 mm was defined as periodontitis [19]. Besides this, ≥2 sites with RABL ≥ 6 mm was also defined as periodontitis [19]. Further, we regarded the CPI code being 3 and 4 as moderate or severe periodontitis, in keeping with the study conducted by Zenthofer et al. [20].

On the base of the diagnostic criteria [21], mild memory impairment (MMI) was approximate to mild cognitive impairment (MCI), so MCI was used instead of MMI in this analysis.

Moreover, the value of MMSE ≤ 20 was described as dementia [22] in our study. In addition, the value of MMSE ≤ 23 [23] or the score of MoCA < 26 [24] was diagnosed as cognitive impairment.

### 2.7. Statistical Analysis

Risk ratio (RR), odds ratio (OR) and 95%CI were calculated to assess the relationship between periodontitis and dementia/cognitive impairment. For continuous outcomes, mean deviation (MD) was used to study the periodontal status of dementia patients. The I^2^ test was used to assess heterogeneity among studies. Subgroup analysis was performed to examine the effects of moderate or severe periodontitis on dementia. Sensitivity analysis was conducted to assess the robustness of results of each meta-analysis. *p* values less than 0.05 were regarded as statistically significant. All results were analyzed using software Review Manager 5.2.

## 3. Results

### 3.1. Included Studies

**Figure 1:**
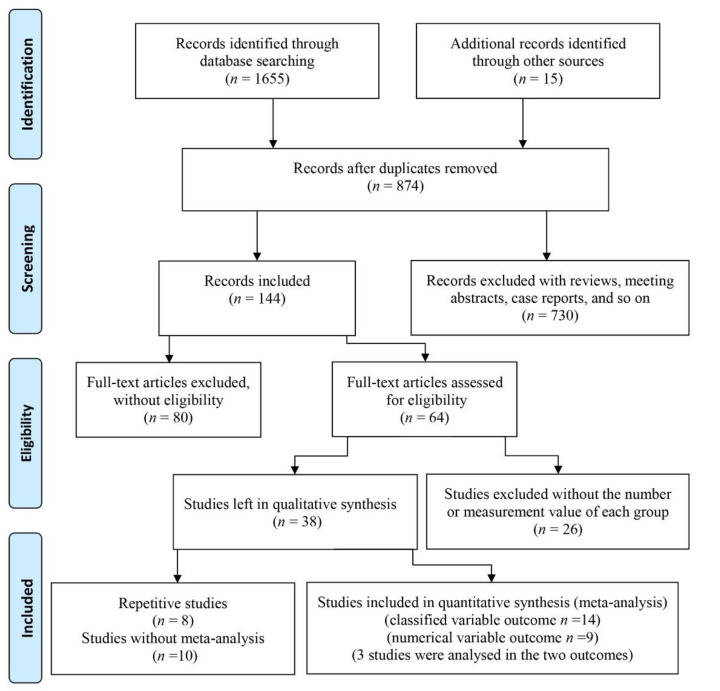
Flow diagram of study selection.

There were 1655 articles obtained by searching online databases and 15 articles obtained by retrieving the references. After excluding duplicated studies, 874 papers remained. Then, after screening abstracts, 253 reviews and letters, 461 papers with abstracts and titles that did not match the theme, and 16 articles without English languages were excluded, 144 papers were left. A total of 80 of these 144 articles were omitted because they only described either periodontal disease or dementia/cognitive impairment in old adults. Among the remaining 64 studies, 26 studies were excluded for lacking a specific number for each group or measurement indexes. Therefore, 38 studies were left. Three papers by the same author analyzed the Fujiwara-kyo Study [21,25,26], and a paper published in December 2010 was included as it captured sufficient data [25]. Similarly, among four other papers [27,28,29,30], only the paper with clear grouping [27] was included. Another two studies were completed by the same hospital [31,32], and only the paper with the larger sample size was included [32]. As the data of the two cohort studies were both from the National Health Insurance Research Database (NHIRD) in Taiwan [33,34], they were not included in the meta-analysis. A study whose outcome was predicted by the Intelligence-Struktur-Test (I-S-T 2000R) was also excluded [35]. Five studies with sole measure indexes of periodontitis disease could not be used in the meta-analysis. To explain this more clearly, two studies described the mean and standard deviation (SD) of five CPI codes [36], and the number of teeth with periodontal pockets ≥ 4 mm [37], respectively. Another two studies assessed cases of periodontal disease with periodontal profile classes [38] and the oral health index [39]. Further, the study conducted by Chen [40] showed the mixing percent calculus, plaque and gingival bleeding. Two papers were omitted which assessed dementia/cognitive impairment with sole indexes, such as the digit symbol test (DST) [41], and the MMSE (where points decreased by 3.00) [42]. A study where the proportion of periodontitis in different groups was based on scores of the digit symbol substitution test (DSST) [43] was excluded, and a study using the mean score of symbol digit substitution test (SDST) [44] in different groups based on periodontitis was also excluded. Finally, 8 case-control studies [27,32,45,46,47,48,49,50], 4 cross-sectional studies [24,25,51,52] and 8 descriptive studies [20,53,54,55,56,57,58,59] were included in this study. The search and screening process of articles are displayed in Figure 1.

### 3.2. Main Characteristics of Included Studies

As shown in Table 1, there were five studies which assessed periodontal status according to the criteria of the WHO with the CPI [20,24,25,48,50]. Further, three studies examined periodontal status by uniting the CPI with other oral health indexes, such as the GI [47,56], PI [47] and the GBI [55]. One diagnosed periodontitis with only PPD [58]. Another three papers used PPD and CAL as their periodontitis indexes [32,52,54]. Except for PPD and CAL, two studies also checked oral status with BOP [49], the PI and the BI [27]. The study performed by Lee recorded PPD and the PI [51]. At the same time, one study only assessed periodontal status with the GI on half-mouth [53]. Two studies used panoramic radiography to assess the history of periodontitis with MABL [45] and RABL [46]. One study diagnosed periodontitis with medical records [57] and the other with unclear criteria [59]. As shown above, the definition of periodontitis varied.

There was diversity in the definition of dementia and cognitive impairment in the included studies. Four studies assessed dementia according to the Diagnostic and Statistical Manual of Mental Disorders, Fourth Edition (DSM-IV) [27,48,51,58] and one study assessed cognitive impairment with DSM-III revised criteria (DSM-III R) [25]. Two studies diagnosed dementia in terms of the National Institute of Neurological and Communicative Disorders and Stroke and the Alzheimer’s Disease and Related Disorders Association (NINCDS-ADRDA) workgroup recommendations [32,54]. The diagnosis of dementia in eight studies was based on medical records [45,47,50,52,53,55,56,57]. Three studies assessed cognitive impairment with the Mini-Mental State Examination-Korean version (MMSE-KC) [46], MoCA test [24] and Clinical Dementia Rating (CDR) along with the MMSE [49], respectively. One study assessed dementia with the MMSE [20], and the assessment was unclear in this study performed by Zhu et al. [59].

### 3.3. Quality Assessment of Eligible Studies

**Figure 2:**
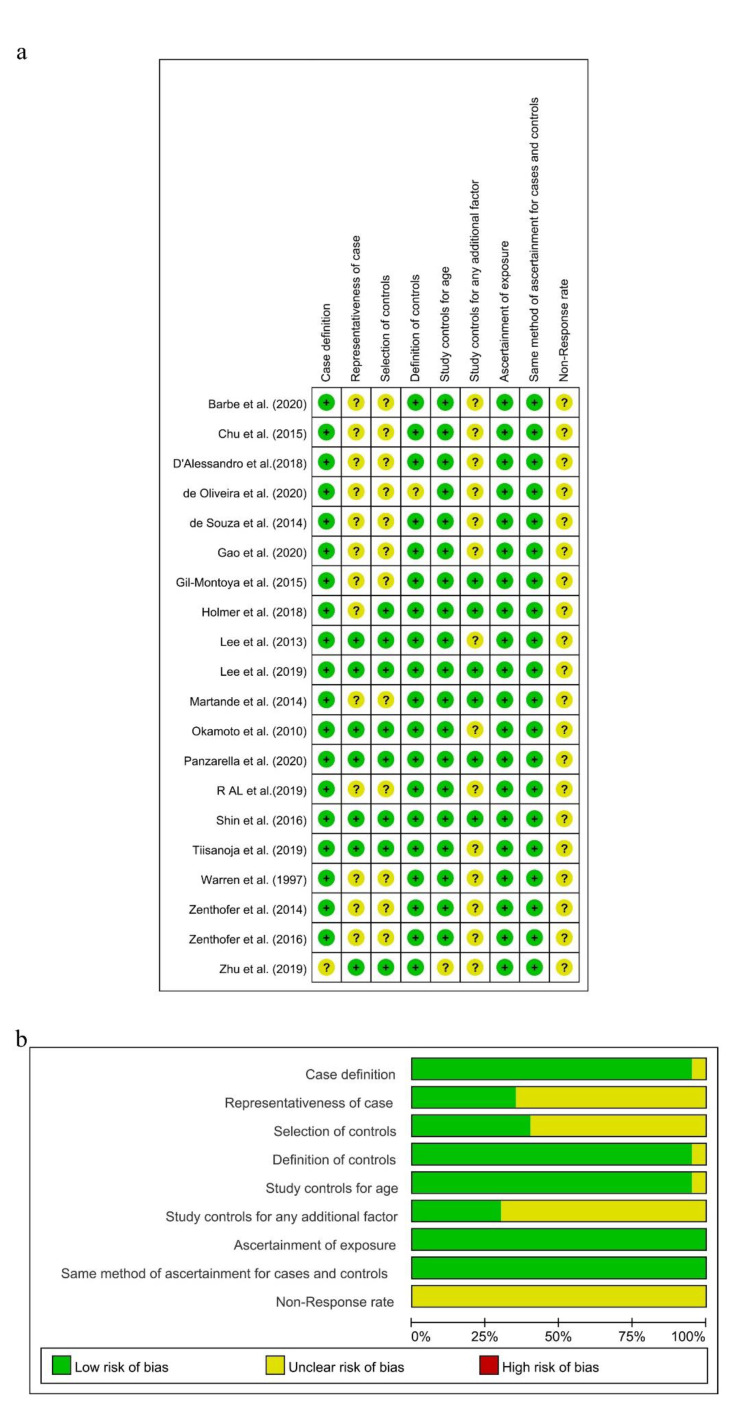
Risk of bias summary (**a**) and risk of bias graph (**b**) for included studies, respectively.

As the diagnostic criteria of AD in the study conducted by Zhu A was unclear [59], there was a potential for case definition bias in terms of the NOS, and the risk of this item presented as “Unclear” in Review Manager. Because all the subjects in these 12 studies were selected from hospitals [20,24,27,32,47,49,50,52,53,54,55,56], there were potential selection biases in the cases and controls. A potential selection bias in the representativeness of the cases was also present in this study, with the dementia group selected from the Karolinska Memory Clinic [45]. Then, the risks of “Representativeness of the Cases” and “Selection of Controls” in these studies were showed as “Unclear” in Review Manager. Both case groups and control groups in these remaining seven studies were selected from communities, and the risks of “Representativeness of the Cases” and “Selection of Control” were judged as “Low”. Depression [60] was controlled in six studies [27,45,46,48,54,57], so the risk of “Study controls for any additional factor” was assessed as “Low”. Meanwhile, this risk in other studies which did not control for depression was assessed as “Unclear”. The non-response rate in all included studies was not obtained or different in two groups, and the risk of “Non-response rate” was assessed as “Unclear.” As shown in Figure 2a,b and Supplementary Table S2, seven studies were assessed to be of high quality with low risk [25,45,46,48,51,57,58], and the others were judged to be of moderate quality with NOS scores of 5, 6, or 4.

### 3.4. Association between Periodontitis and Cognitive Impairment

Six studies were chosen to assess the association between periodontitis and cognitive impairment [24,25,27,45,46,59]. As the number of included studies was less than 10, reporting bias was not performed.

**Figure 3:**
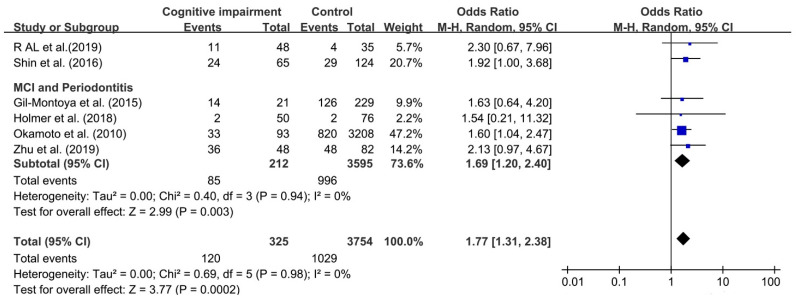
Forest plot of association between cognitive impairment and periodontitis.

Although I^2^ was 0%, a random effect model was still chosen to analyze this association because of obvious clinical heterogeneity in this comparison. We found that the association between periodontitis and cognitive impairment was significant, with OR 1.77 (95%CI, 1.31–2.38), which meant that cognitive impairment among individuals with periodontitis was increased by 77% over individual without periodontitis (as shown in Figure 3). The OR of the relationship between MCI and periodontitis was 1.69 (95%CI, 1.20–2.40).

Sensitivity analysis was conducted by excluding studies in turn, and the results were consistent (results not shown).

### 3.5. Relationship between Periodontitis and Dementia

**Figure 4:**
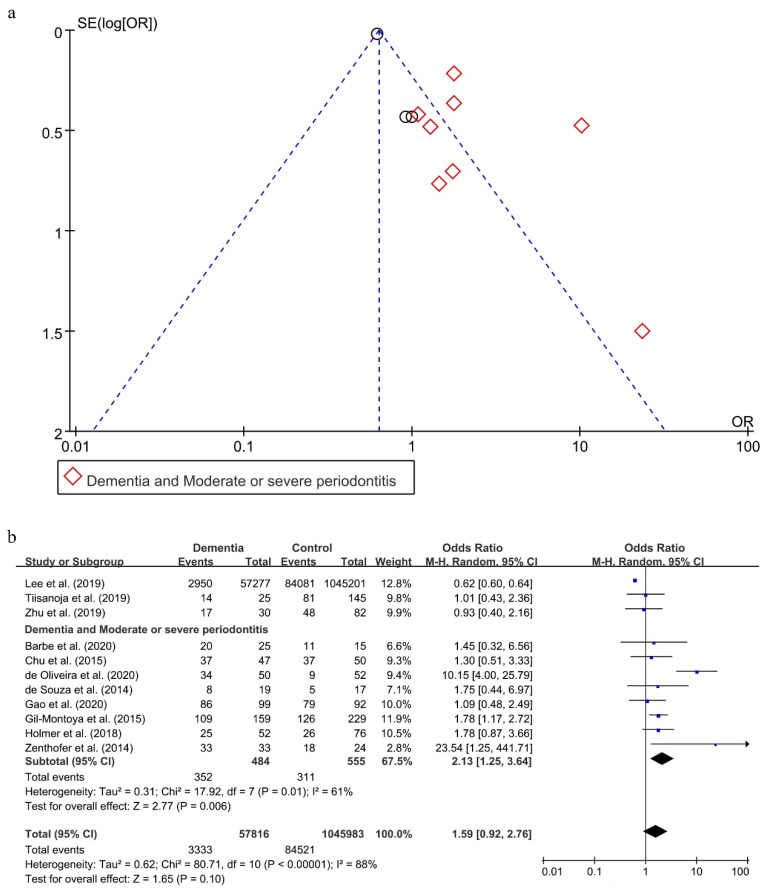
Funnel Plot (**a**) and forest plot (**b**) of the relationship between dementia and periodontitis.

As shown in Figure 4a, a reporting bias was found in these 11 included studies [20,27,32,45,47,49,50,52,57,58,59].

Without considering the severity of periodontitis, there was an obvious statistical heterogeneity, with I^2^ being 88% in this analysis, and a random effect model was chosen to analyze this association. There was no statistical significance in the effect of periodontitis on dementia (OR = 1.59; 95%CI, 0.92–2.76) (Figure 4b). When subgroups were grouped based on the severity of periodontitis, the statistical heterogeneity declined. The OR of the association between moderate or severe periodontitis and dementia was 2.13 (95%CI, 1.25–3.64), meaning that individuals with moderate or severe periodontitis were 2.13 times more likely to suffer from dementia than persons without moderate or severe periodontitis.

The two results were stable in the process of sensitivity analysis. Interestingly, only when eliminating both the studies conducted by Lee et al. [57] and de Oliveira et al. [49], reporting bias disappeared (shown in Supplementary Figure S3a) and the statistical heterogeneity decreased with I^2^ being 0% (shown in Supplementary Figure S3b). Moreover, the result between periodontitis and dementia also reversed (OR = 1.48; 95%CI, 1.13–1.94).

### 3.6. Periodontal Status in Dementia Patients

Seven clinical indexes, including the GI, PI, CPI, PPD, CAL and the GBI, were used to assess the periodontal status among dementia patients in nine studies [27,47,48,49,51,53,54,55,56]. Because the number of studies in each comparison was less than 10, reporting bias was not conducted.

**Figure 5:**
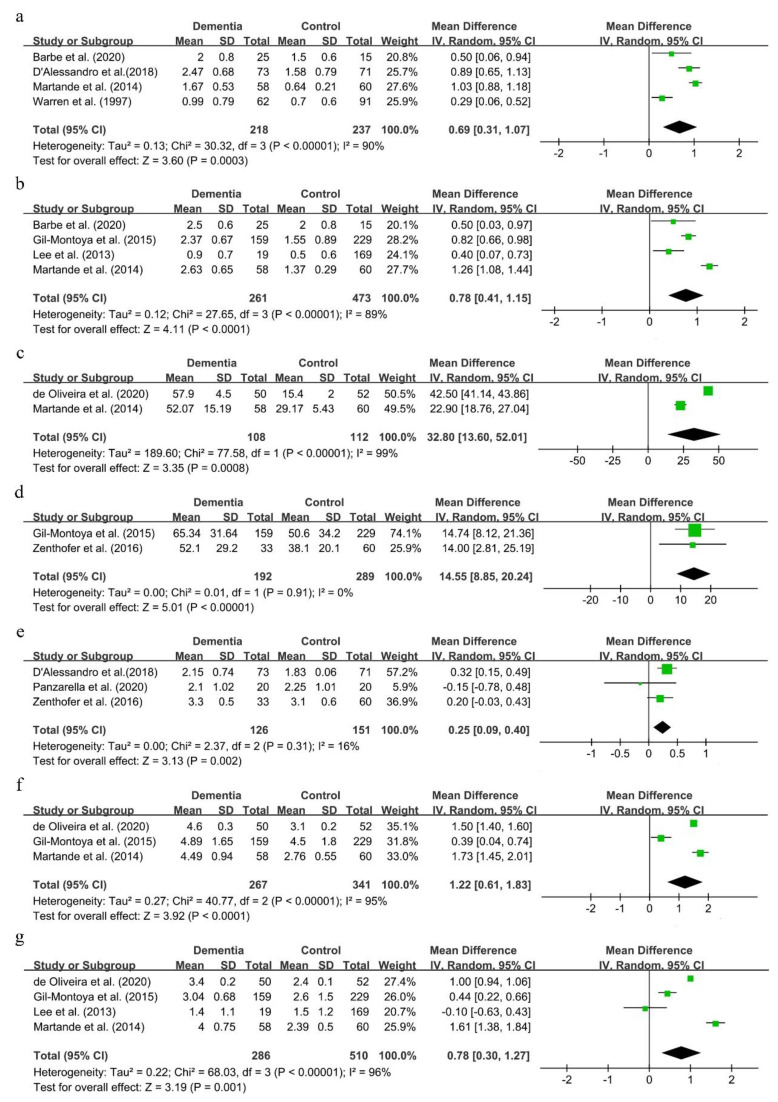
Forest plot of periodontal status in dementia patients (**a**). GI; (**b**). PI; (**c**). BOP; (**d**). GBI; (**e**). CPI; (**f**). CAL; (**g**). PPD.

The mean deviations of the GI and PI were 0.69 (95%CI, 0.31–1.07) and 0.78 (95%CI, 0.41–1.15), respectively (shown in Figure 5a,b). Though there was great statistical heterogeneity in the two comparisons, results remained consistent in sensitivity analysis (results not shown).

As shown in Figure 5c–f, the mean deviations of BOP, the GBI, CPI and CAL were 32.8 (95%CI, 13.60–52.01), 14.55 (95%CI, 8.85–20.24), 0.25 (95%CI, 0.09–0.40) and 1.22 (95%CI, 0.61–1.83), respectively. These suggested that dementia patients showed a worse periodontal status than people with normal cognitive function.

The MD of PPD was 0.78 (95%CI, 0.30–1.27) (shown in Figure 5g). This result was not consistent in sensitivity analysis. Either the studies conducted by de Oliveira et al. [49] or Martande et al. [54] were excluded, the results of PPD reversed, and the statistical heterogeneity obviously remained in this process (shown in Supplementary Figure S4a,b).

## 4. Discussion

The results of case-control studies showed a relationship between periodontitis and cognitive impairment. These results were consistent in the process of sensitivity analysis, which was also consistently found in many studies [41,43,44,61,62]. For example, a study showed that the multivariable adjusted OR between low MMSE score and periodontal disease was 2.21 (95%CI, 1.01–4.84), and it concluded that periodontal disease was significantly associated with cognitive impairment [62]. Further, two cohort studies also supported this result [42,63]. For instance, a 6-year follow-up cohort study described a statistically significant association between the prevalence of periodontitis and cognitive decline [42]. Moreover, the relationship between MCI and periodontitis was also stable in the process of sensitivity analysis, which was demonstrated by the cohort study where periodontal disease was modestly associated with incident MCI [38]. Thus, we concluded that periodontitis was associated with cognitive impairment and MCI in this meta-analysis.

Though the result reversed when sensitivity analysis was applied, there might not be a relationship between periodontitis and dementia in this meta-analysis, which was also obtained in some studies [57,58]. When periodontitis was defined as more than 1 tooth with periodontal pockets greater than 4mm, the relative risk of periodontitis and dementia was 1.54 without significant difference [58]. However, some cohort studies [33,34,64,65] supported the effect of periodontitis on dementia. An analysis of IgG antibody levels in seven oral bacterial species associated with periodontitis also suggested that periodontal disease may contribute to AD onset/progression [66]. However, the severity of periodontitis and the percent of moderate or severe periodontitis cases in these studies were unclear, and thus we could not fairly assess the relationship between dementia and periodontitis; periodontitis, then, might not contribute to dementia in this meta-analysis.

We found the relationship between moderate or severe periodontitis and dementia was consistent in sensitivity analysis. The main reason was that there were deep periodontal pockets in cases of moderate or severe periodontitis [67]. As is well known, the deeper these pockets are, the more inflammatory cytokines and periodontal pathogens, which contribute to dementia, there are [18,68]. Moreover, lipopolysaccharide derived from *Porphyromonas gingivalis* was detected in the brains of AD patients [69] and activated nerve cells in the hippocampi of mice [70]. Furthermore, microglial cells can respond to this pathogen with inflammation [71,72]. Thus, given the results of our current analysis and previous laboratory studies, subjects with moderate or severe periodontitis were at greater risk of developing dementia.

The results of our meta-analysis showed that dementia patients had a worse periodontal status, which was also consistent with some studies [36,37]. When compared to the control group, AD patients exhibited fewer periodontal healthy sextants (0.1 ± 0.4 vs. 1.4 ± 2.2) in a case-control study [36]. As is well known, dementia interferes with daily activities gradually, so it is difficult for dementia patients and their caregivers to conduct oral hygiene effectively, especially for severe dementia patients. Thus, it is reasonable that dementia patients had a worse periodontal status. The reason why the result of the PPD index reversed in sensitivity analysis was that there was no statistical difference in the PPD index between dementia and cognitively intact persons in the two included papers [27,51]. Thus, whether the PPD index in dementia patients was significantly greater than in cognitively intact persons needs further study. Briefly, periodontal status in dementia patients was much poorer in comparison with cognitively intact participants.

Though this was the first meta-analysis which determined that moderate or severe periodontitis could be associated with dementia, there were some limitations. First, most included studies lacked related details about dementia, so we could not assess the severity and types of dementia. Second, there was great clinical heterogeneity in each included study, such as the various assessments of periodontitis and cognition status used, and the different sensibility of diagnosis indexes. For a study with a small sample size, there would be some potential of bias in the CPI, with the constituent ratio of periodontal disease likely declining [73]. For studies with a large sample size, the CPI and medical history according to the International Classification of Diseases, 11th Revision [74] might be the best choice. Moreover, panoramic radiography could be a good choice to assess periodontal disease among dementia patients if it can be mobile. For dementia and cognitive impairment, the use of the MMSE scale [22] and medical history according to the International Classification of Diseases, 11th Revision [74] should be the most common choice. Third, as only case-control and cross-sectional studies were included, we could not assess the causal relationship between periodontitis and cognition status. Thus, far more high-quality cohort studies should be conducted to observe the effect of periodontitis on dementia/cognitive impairment.

## 5. Conclusions

In this meta-analysis, we concluded that periodontitis was associated with cognitive impairment, and subjects with moderate or severe periodontitis were at greater risk of developing dementia. Further, we found that dementia patients had a poor periodontal status. However, further well-designed studies, especially cohort studies, should be conducted to confirm this relationship between periodontal disease and dementia/cognitive impairment.

## Figures and Tables

**Table 1:** Characteristics of studies in qualitative synthesis.

First Author-Year	Age (Years)	Sample Size	Cognition Status Criteria	PD Criteria	Conclusion
**Articles included in the quantitative analysis**					
Barbe et al. (2020) [47]	82	40	medical records	CPITN	There was no relationship between periodontitis and dementia (*p* = 0.705).
Chu et al. (2015) [50]	≥60	97	medical records	CPI	There was no significant difference in the prevalence of advanced periodontal disease (CPI ≥ 3) between the dementia and control group (*p* = 0.64).
D’Alessandro et al. (2018) [56]	>65	144	medical records	CPI, GI	AD patients presented numbers of CPI, and GI was significantly higher (*p* ≤ 0.005).
de Oliveira et al. (2020) [49]	71.17	102	CDR and MMSE	PPD, CAL	AD patients had greater CAL than controls. Periodontitis was a variable most likely associated with AD (*p* < 0.001).
de Souza et al. (2014) [32]	≥59	36	NINCDS-ADRDA	PPD, CAL	A higher prevalence of periodontal infections (*p* = 0.002) was observed in the AD group compared to the control group.
Gao et al. (2020) [52]	≥65	187	medical records	PPD, LoA	There was no significant difference of periodontal status observed in the dementia group compared to the control group.
Gil-Montoya et al. (2015) [27]	>50	388	DSM-IV and NINCDS-ADRDA	CAL	A statistically significant association was observed between CAL and cognitive impairment after controlling for confounding factors (*p* = 0.049).
Holmer et al. (2018) [45]	≥50	128	medical records	MABL	Marginal periodontitis was associated with early cognitive impairment and AD.
Lee et al. (2013) [51]	≥70	188	DSM- IV	PPD, PI	There was no significant difference of pocket depth and plaque index observed.
Lee et al. (2019) [57]	≥65	1102478	medical records	medical records	There was a significant relationship between periodontitis and dementia, except for the group of men aged ≥81 years.
Martande et al. (2014) [54]	≥50	118	NINCDS-ADRDA	PPD, CAL	The periodontal health status of individuals with AD deteriorated with disease progression and was closely related to their cognitive function.
Okamoto et al. (2010) [25]	≥65	3456	DSM-III R	CPI	There was a significant relationship between periodontitis and MMI (*p* = 0.043).
Panzarella et al. (2020) [48]	81.15	60	DSM-IV	CPI	The scores of the CPI did not statistically differ between AD patients and control group.
R et al. (2019) [24]	≥ 60	83	MoCA	CPI	No statistical significant correlation with regard to periodontal disease and MoCA test scores (*p* = 0.319).
Shin et al. (2016) [46]	69.04	189	MMSE-KC	RABL	Periodontitis was independently associated with cognitive impairment after controlling for various confounders.
Tiisanoja et al. (2019) [58]	80.9	170	DSM-IV	PPD	Periodontal disease and stomatitis were associated, although non-statistically, with AD and dementia.
Warren et al. (1997) [53]	80.9	118	medical records	GI	Those with severe dementia had poorer gingival health and oral hygiene.
Zenthofer et al. (2014) [20]	80.9 ≥ 54	57	MMSE	CPITN	Mean CPITN of participants in the dementia group was significantly worse than those of participants in the non-dementia group (*p* < 0.001).
Zenthofer et al. (2016) [55]	≥54	93	medical records	GBI, CPITN	In bivariate testing, participants with dementia had a significantly lower GBI (*p* < 0.05), and a lower CPITN (*p* < 0.01) at follow-up.
Zhu et al. (2019) [59]	64.06	112	unclear	unclear	Executive function, language and short-term memory of early cognitive decline were associated with periodontal disease.
**Articles excluded in the quantitative analysis**					
Aragon et al. (2018) [36]	72.38	106	McKhann et al. diagnosed criteria	CPI	After taking into account the influence of age, Alzheimer’s patients had worse oral health (caries and periodontal disease).
Cestari et al. (2016) [31]	≥56	65	NINCDS-ADRDA	PPD, CAL	There were no differences in periodontal indexes among groups.
Chen et al. (2013) [40]	≥50	700	MMSE	Calculus/PI/GBI (%)	Demented participants presented with heavy plaque/calculus or severe gingival bleeding, significantly more than that in non-impaired group (*p*< 0.01).
Chen et al. (2017) [33]	≥50	27963	ICD-9-CM	ICD-9-CM	10-year chronic periodontitis exposure was associated with a 1.707-fold increase in the risk of developing AD.
Demmer et al. (2020) [38]	63	8275	DSM-V	Periodontal Profile Class	Periodontal disease was modestly associated with incident MCI and dementia in a community-based cohort of black and white participants.
Gil-Montoya et al. (2020) [30]	76.8	309	DSM-IV and NINCDS-ADRDA	CAL	Systemic inflammation derived from periodontal disease plays a relevant role in the aetiology of cognitive impairment.
Gil-Montoya et al. (2017) [28]	≥51	564	DSM-IV and NINCDS-ADRDA	BI, PI	Gingival inflammation is independently associated with cognitive impairment, even at its earliest stage.
Gil-Montoya et al. (2017) [29]	≥51	288	DSM-IV and NINCDS-ADRDA	CAL	Periodontitis may be a modulating variable of the association between Aβ and cognitive impairment.
Kamer et al. (2012) [41]	70	152	DST	PI	Subjects with PI had significantly lower adjusted mean DST scores compared to subjects without PI.
Nilsson et al. (2018) [42]	≥60	566	MMSE	MABL	A statistically significant association between prevalence of periodontitis and cognitive decline after adjustments of confounding factors.
Okamoto et al. (2010) [26]	≥65	2646	MMSE	CPI	No significant differences were found in CPI code between the two groups.
Okamoto et al. (2017) [21]	≥65	471	MMSE	CPI	No significant differences were found in CPI code between the two groups.
Ribeiro et al. (2012) [39]	≥59	60	DSM-IV	OHI	Elderly subjects with AD had poorer oral health than those without the disease.
Sorensen et al. (2018) [35]	56	193	Intelligence-Struktur-Test	PPD	The two groups did not differ significantly with respect to the presence of periodontitis.
Sung et al. (2019) [44]	≥20	4663	SRTT, SDST, SDLT	PPD, CAL	Periodontal status was associated with cognitive impairment in a nationally representative sample of US adults.
Syrjala et al. (2012) [37]	82	180	DSM-IV	PPD	Dementia patients had an increased likelihood of having teeth with deep periodontal pockets, compared with non-demented persons.
Tzeng et al. (2016) [34]	≥20	8828	DSM-IV	ICD-9-CM	Patients with chronic periodontitis and gingivitis have a higher risk of developing dementia.
Yu et al. (2008) [43]	70.4	803	DSST	BOP	Higher cognitive function was associated with lower odds of periodontal disease.

Abbreviations: ¯, mean; AD, Alzheimer’s disease; BI, Bleeding Index; BOP, Bleeding on Probing; CAL, Clinical Attachment Loss; CDR, Clinical Dementia Rating; CPI, Community Periodontal Index; CPITN, Community Periodontal Index of Treatment Needs; DSM-IV, Diagnostic and Statistical Manual, Fourth edition; DST, Digit Symbol Test; DSST, Digit Symbol Substitution Test; GBI, gingival bleeding index; GI, gingival index; ICD-9-CM, International Classification of Diseases, Ninth Revision, Clinical Modification; LoA, Loss of Attachment; MMSE, Mini-mental State Examination; MABL, Marginal alveolar bone loss; MCI, mild cognitive impairment; MMI, mild memory impairment; MMSE-KC, Mini-mental State Examination-Korean version; MoCA, Montreal Cognitive Assessment; NINCDS-ADRDA, National Institute of Neurological and Communicative Disorders and Stroke and the Alzheimer’s Disease and Related Disorders Association; OHI, oral health index; PD, periodontal disease; PI, Plaque Index; PPD, Probing Pocket Depth; RABL, Radiographic Alveolar Bone Loss; SDLT, Serial Digit Learning Test; SDST, Symbol Digit Substitution Test; SRTT, Simple Reaction Time Test.

## Data Availability

All data generated or analyzed during this study are included in this published article (and its Supplementary Files).

## Supplementary Materials

The following are available online at https://www.mdpi.com/article/10.3390/ijerph18136823/s1, Search strategies S1: Search strategies in different databases, Table S2: Quality assessment of case-control studies according to the NOS, Figure S3: Funnel Plot (a) and forest plot (b) of sensitivity analysis in studies about dementia and periodontitis, Figure S4: Sensitivity analysis in studies about periodontal status in dementia patients.
