# Supplementary material for: The Effect of Periodontitis on Dementia and Cognitive Impairment: A Meta-Analysis
Source: Int J Environ Res Public Health. 2021 Jun 25;18(13):6823. doi: 10.3390/ijerph18136823 (PMC8297088; doi:10.3390/ijerph18136823)
Supplement: Supplementary file 1 [file ijerph-18-06823-s001.zip › supplement.pdf]

The effect of periodontitis on dementia and cognitive impairment: a meta-analysis

Haiying Guo[1], Shuli Chang[1], Xiaoqin Pi[1], Fang Hua[1,2], Han Jiang[1], Chang Liu[1*], Minquan Du[1*]

1. The State Key Laboratory Breeding Base of Basic Science of Stomatology (Hubei-MOST) & Key Laboratory of Oral Biomedicine Ministry of Education, School & Hospital of Stomatology, Wuhan University, Wuhan, Hubei, China
2. Center for Evidence-Based Stomatology, School & Hospital of Stomatology, Wuhan University, Wuhan, Hubei, China

Key words: periodontal disease; periodontitis; dementia; cognitive impairment; meta-analysis

---

*Corresponding author: The State Key Laboratory Breeding Base of Basic Science of Stomatology (Hubei-MOST) & Key Laboratory of Oral Biomedicine Ministry of Education, School & Hospital of Stomatology, Wuhan University, 237 Luoyu Road, Wuhan, Hubei, China

E-mail: duminquan@whu.edu.cn (M. Du), liuc0728@whu.edu.cn (C. Liu)

Tel: 027-87686227

Supplement

Supplementary search strategies

S1. Search strategies in different databases

1.1 Search strategy in Web of science

#1 “periodontal disease” OR “periodontitis” OR “gingivitis” OR “oral health” OR “oral hygiene”

#2 “dementia” OR “Alzheimer’s disease” OR “cognitive decline” OR “cognitive impairment”

Search query: #1 AND #2

1.2 Search strategy in EMBASE

#1 'periodontal disease' OR 'periodontitis' OR 'gingivitis' OR 'oral health' OR 'oral hygiene'

#2 'dementia' OR 'Alzheimer disease' OR 'cognitive decline' OR 'cognitive defect'

Search query: #1 AND#2

Supplementary Table S2. Quality assessment of case-control studies according to the NOS

1.1 R AL, Alzahrani S, Alhefdhi R, Altamimi A, Alfadhel A, Alshareef A, Aldawsari B, Sonbol S, Alsubaie F, Alwahibi A, A AL-S (2019) The relation between teeth loss and cognitive decline among Saudi population in the city of Riyadh: A pilot study.

**Cross sectional study**	A. LFotawi R-2019	
**Selection**<br>1) Is the case definition adequate<br>a) yes, with independent validation*<br>b) yes, eg record linkage or based on self-reports<br>c) no description	Participants with decreased cognitive state were those with MoCA test scores below the usual cutoff level (<26).	a*
2) Representativeness of the cases<br>a) consecutive or obviously representative series of cases*<br>b) potential for selection biases or not stated	A pilot cross sectional survey targeting geriatric population 60-years-old was undertaken at different centers in Riyadh City, Kingdom of Saudi Arabi	b
3) Selection of Controls<br>a) community controls*<br>b) hospital controls<br>c) no description	As stated above.	b
4) Definition of Controls<br>a) no history of disease (end point)*<br>b) no description of source	Participants with decreased cognitive state were those with MoCA test scores below the usual cutoff level (<26).	a*
**Comparability**<br>5) Comparability of cases and controls on the basis of the design or analysis<br>a) study controls for age *	Geriatric population 60-years-old was selected.	a*

b) study controls for any additional factor *		
**Exposure**<br>6) Ascertainment of exposure<br>a) secure record (eg surgical records) *<br>b) structured interview where blind to case/control status*<br>c) interview not blinded to case/control status<br>d) written self-report or medical record only<br>e) no description	Six measurements per tooth was taken to assess the average pocket depth (two teeth in each quadrant) using a sterile periodontal probes. Periodontal condition was examined in the participants using CPI.	a*
7) Same method of ascertainment for cases and controls<br>a) yes*<br>b) no	As stated above.	a*
8) Non-Response rate<br>a) same rate for both groups *<br>b) non respondents described<br>c) rate different and no designation secure record (eg surgical records)		c
Risk of bias		5/9(Moderate)
Finally quality assessment		Moderate
* point		

1.2 Barbe AG, Kupeli LS, Hamacher S, Noack MJ (2020) Impact of regular professional toothbrushing on oral health, related quality of life, and nutritional and cognitive status in nursing home residents.

**Case-control study**	Barbe AG-2020	
**Selection**<br>1) Is the case definition adequate<br>a) yes, with independent validation*<br>b) yes, eg record linkage or based on self-reports<br>c) no description	Residents with a dementia diagnosis or a diagnosis of cognitive impairment were documented by a yes/no answer if the diagnosis was given by a neurologist.	a*
2) Representativeness of the cases<br>a) consecutive or obviously representative series of cases*<br>b) potential for selection biases or not stated	Nursing home residents at the St. Elisabeth senior residence (Bornheim, Germany) guardians were asked to participate in our study.	b
3) Selection of Controls<br>a) community controls*<br>b) hospital controls<br>c) no description	As stated above.	b
4) Definition of Controls<br>a) no history of disease (end point)*<br>b) no description of source	Mental State Examination (MMSE) were chosen to assess the cognitive abilities.	a*
**Comparability**<br>5) Comparability of cases and controls on the basis of the design or analysis<br>a) study controls for age *<br>b) study controls for any additional factor *	There was no significance in age between cases and controls with P being 0.705.	a*

**Exposure**<br>6) Ascertainment of exposure<br>a) secure record (eg surgical records) *<br>b) structured interview where blind to case/control status*<br>c) interview not blinded to case/control status<br>d) written self-report or medical record only<br>e) no description	The periodontal status was measured according to the community periodontal index of treatment needs (CPITN).	a*
7) Same method of ascertainment for cases and controls<br>a) yes*<br>b) no	As stated above.	a*
8) Non-Response rate<br>a) same rate for both groups *<br>b) non respondents described<br>c) rate different and no designation secure record (eg surgical records)		c
Risk of bias		5/9(Moderate)
Finally quality assessment		Moderate
* point		

1.3 Chu CH, Ng A, Chau AM, Lo EC (2015) Oral health status of elderly Chinese with dementia in Hong Kong

**Case-control study**	Chu CH-2015	
**Selection**<br>1) Is the case definition adequate<br>a) yes, with independent validation*<br>b) yes, eg record linkage or based on self-reports<br>c) no description	The diagnosis and the stage of dementia were recorded.	a*
2) Representativeness of the cases<br>a) consecutive or obviously representative series of cases*<br>b) potential for selection biases or not stated	Recruitment of participants was carried out in the day-care centres of the Hong Kong Alzheimer's Disease Association and St. James' Settlement Kin Chi Dementia Care Support Service Center.	b
3) Selection of Controls<br>a) community controls*<br>b) hospital controls<br>c) no description	A control group was recruited from the registered list of Chinese people who had attended Prince Philip Dental Hospital .	b
4) Definition of Controls<br>a) no history of disease (end point)*<br>b) no description of source	Matching the gender and age, the same number of generally healthy elderly without dementia were recruited to act as the control group.	a*
**Comparability**<br>1) Comparability of cases and controls on the basis of the design or analysis<br>a) study controls for age *<br>b) study controls for any additional factor *	Aged 60 or older.	a*

**Exposure**<br>1) Ascertainment of exposure<br>a) secure record (eg surgical records) *<br>b) structured interview where blind to case/control status*<br>c) interview not blinded to case/control status<br>d) written self-report or medical record only<br>e) no description	The examination assessed periodontal status using the community periodontal index (CPI).	a*
2) Same method of ascertainment for cases and controls<br>a) yes*<br>b) no	As stated above.	a*
3) Non-Response rate<br>a) same rate for both groups *<br>b) non respondents described<br>c) rate different and no designation secure record (eg surgical records)	Seventeen percent (n = 10) of people with dementia were edentulous, as were 14% (n = 8) in the control group.	c
Risk of bias		5/9(Moderate)
Finally quality assessment		Moderate
* point		

1.4 D'Alessandro G, Costi T, Alkhamis N, Bagattoni S, Sadotti A, Piana G (2018) Oral Health Status in Alzheimer's Disease Patients: A Descriptive Study in an Italian Population

Cross-sectional study	D'Alessandro G-2018	
**Selection**<br>1) Is the case definition adequate<br>a) yes, with independent validation*<br>b) yes, eg record linkage or based on self-reports<br>c) no description	The selection criteria for the study sample were a confirmed diagnosis of AD.	a*
2) Representativeness of the cases<br>a) consecutive or obviously representative series of cases*<br>b) potential for selection biases or not stated	AD patients were recruited from a public elderly institute “Istituto Giovanni XXIII” in Bologna and a daytime recreational center in Rimini, Italy.	b
3) Selection of Controls<br>a) community controls*<br>b) hospital controls<br>c) no description		c
4) Definition of Controls<br>a) no history of disease (end point)*<br>b) no description of source	A control group was used for comparison with the following inclusion criteria: negative medical history for dementia.	a*
**Comparability**<br>5) Comparability of cases and controls on the basis of the design or analysis<br>a) study controls for age *<br>b) study controls for any additional factor *	All participants were older than 65 years.	a*

**Exposure**<br>6) Ascertainment of exposure<br>a) secure record (eg surgical records) *<br>b) structured interview where blind to case/control status*<br>c) interview not blinded to case/control status<br>d) written self-report or medical record only<br>e) no description	The CPI and the GI of Silness-Löe were recorded as recommended by the codes and criteria of the WHO.	a*
7) Same method of ascertainment for cases and controls<br>a) yes*<br>b) no	As stated above.	a*
8) Non-Response rate<br>a) same rate for both groups *<br>b) non respondents described<br>c) rate different and no designation secure record (eg surgical records)		c
Risk of bias		5/9(Moderate)
Finally quality assessment		Moderate
* point		

1.5 de Oliveira Araújo R, Villoria GEM, Luiz RR, Esteves JC, Leão ATT, Feres-Filho EJ (2020) Association between periodontitis and Alzheimer's disease and its impact on the self-perceived oral health status: a case-control study.

**Case-control study**	de Oliveira R-2020	
**Selection**<br>1) Is the case definition adequate<br>a) yes, with independent validation*<br>b) yes, eg record linkage or based on self-reports<br>c) no description	Only those with mild or moderate AD, according to the Clinical Dementia Rating (CDR = 1 or 2, respectively) and Mini-mental State Examination (MMSE) scores ≥ 13, were included	a*
2) Representativeness of the cases<br>a) consecutive or obviously representative series of cases*<br>b) potential for selection biases or not stated	This case-control study was conducted in Brazil, at the Center of Alzheimer's Disease of the Psychiatric Institute of the Federal University of Rio de Janeiro.	b
3) Selection of Controls<br>a) community controls*<br>b) hospital controls<br>c) no description	Controls were recruited from family caregivers and were proportionally matched to cases by the variables age and sex.	b
4) Definition of Controls<br>a) no history of disease (end point)*<br>b) no description of source		b
**Comparability**<br>5) Comparability of cases and controls on the basis of the design or analysis<br>a) study controls for age *<br>b) study controls for any additional factor *	Controls were proportionally matched to cases by the variables age and sex.	a*

**Exposure**<br>6) Ascertainment of exposure<br>a) secure record (eg surgical records) *<br>b) structured interview where blind to case/control status*<br>c) interview not blinded to case/control status<br>d) written self-report or medical record only<br>e) no description	The clinical diagnosis of moderate or severe periodontitis was based on the presence of two or more interproximal sites with PD ≥ 5 mm and CAL ≥ 5 mm, not on the same tooth, which bled after probing	a*
7) Same method of ascertainment for cases and controls<br>a) yes*<br>b) no	As stated above.	a*
8) Non-Response rate<br>a) same rate for both groups *<br>b) non respondents described<br>c) rate different and no designation secure record (eg surgical records)	41 subjects in 352 Case group and 21 subjects in 132 Control group did not respond invitation.	c
Risk of bias		4/9(Moderate)
Finally quality assessment		Moderate
* point		

1.6 de Souza Rolim T, Fabri GM, Nitrini R, Anghinah R, Teixeira MJ, de Siqueira JT, Cestari JA, de Siqueira SR (2014) Oral infections and orofacial pain in Alzheimer's disease: a case-control study.

**Case-control study**	de Souza Rolim T-2014	
**Selection**<br>1) Is the case definition adequate<br>a) yes, with independent validation*<br>b) yes, eg record linkage or based on self-reports<br>c) no description	Patients that met the NINCDS-ADRDA criteria for AD classified as mild by the Mini-Mental Status Exam (MMSE, score from 18 to 26) were enrolled in this study.	a*
2) Representativeness of the cases<br>a) consecutive or obviously representative series of cases*<br>b) potential for selection biases or not stated	Patients were recruited from the Cognitive Neurology and Behavior Group of the Neurology Department of the School of Medicine of the University of Sao Paulo, Brazil.	b
3) Selection of Controls<br>a) community controls*<br>b) hospital controls<br>c) no description		c
4) Definition of Controls<br>a) no history of disease (end point)*<br>b) no description of source	The control group comprised 30 elderly without AD.	a*
**Comparability**<br>5) Comparability of cases and controls on the basis of the design or analysis<br>a) study controls for age *<br>b) study controls for any additional factor *	Aged 59 or older.	a*

**Exposure**<br>6) Ascertainment of exposure<br>a) secure record (eg surgical records) *<br>b) structured interview where blind to case/control status*<br>c) interview not blinded to case/control status<br>d) written self-report or medical record only<br>e) no description	Periodontal evaluation with probes to determine gingival bleeding index (GBI), probing pocket depth (PPD), the clinical attachment level (CAL).	a*
7) Same method of ascertainment for cases and controls<br>a) yes*<br>b) no	As stated above.	a*
8) Non-Response rate<br>a) same rate for both groups *<br>b) non respondents described<br>c) rate different and no designation secure record (eg surgical records)	Completely edentulous were different in two groups.	c
Risk of bias		5/9(Moderate)
Finally quality assessment		Moderate
* point		

1.7 Gao SSQ, Chen KJY, Duangthip D, Lo ECM, Chu CH (2020) The Oral Health Status of Chinese Elderly People with and without Dementia: A Cross-Sectional Study

**Cross-sectional study**	Gao SS-2020	
**Selection**<br>1) Is the case definition adequate<br>a) yes, with independent validation*<br>b) yes, eg record linkage or based on self-reports<br>c) no description	The dementia status (yes/no) of the participants were collected from their medical record saved by the daycare centers.	a*
2) Representativeness of the cases<br>a) consecutive or obviously representative series of cases*<br>b) potential for selection biases or not stated	Eight elderly daycare centers in Hong Kong were invited to join this study.	b
3) Selection of Controls<br>a) community controls*<br>b) hospital controls<br>c) no description	As stated above.	b
4) Definition of Controls<br>a) no history of disease (end point)*<br>b) no description of source	The dementia status (yes/no) of the participants were collected from their medical record saved by the daycare centers.	a*
**Comparability**<br>5) Comparability of cases and controls on the basis of the design or analysis<br>a) study controls for age *<br>b) study controls for any additional factor *	Elderly people were aged 65 years or older.	a*

**Exposure**<br>6) Ascertainment of exposure<br>a) secure record (eg surgical records) *<br>b) structured interview where blind to case/control status*<br>c) interview not blinded to case/control status<br>d) written self-report or medical record only<br>e) no description	The dentists determined the periodontal status of the elderly with the CPI examination. The full dentition was divided into six sextants. Gingival bleeding, periodontal pocket, and loss of attachment were recorded by assessing the index teeth in each sextan.	a*
7) Same method of ascertainment for cases and controls<br>a) yes*<br>b) no	As stated above.	a*
8) Non-Response rate<br>a) same rate for both groups *<br>b) non respondents described<br>c) rate different and no designation secure record (eg surgical records)		b
Risk of bias		5/9(Moderate)
Finally quality assessment		Moderate
* point		

1.8 Gil-Montoya JA, Sanchez-Lara I, Carnero-Pardo C, Fornieles F, Montes J, Vilchez R, Burgos JS, Gonzalez-Moles MA, Barrios R, Bravo M (2015) Is periodontitis a risk factor for cognitive impairment and dementia? A case-control study.

**Case-control study**	Gil-Montoya JA-2015	
**Selection**		
1) Is the case definition adequate<br>a) yes, with independent validation*<br>b) yes, eg record linkage or based on self-reports<br>c) no description	All were assessed by a neurologist expert in dementia with a guided anamnesis, general/neurological examination, and extensive neuropsychological assessment.	a*
2) Representativeness of the cases<br>a) consecutive or obviously representative series of cases*<br>b) potential for selection biases or not stated	Cases were recruited from the Neurology Departments of two hospitals.	b
3) Selection of Controls<br>a) community controls*<br>b) hospital controls<br>c) no description	Controls were recruited from among individuals visiting the primary healthcare center for reasons other than a dental or neurological problem.	b
4) Definition of Controls<br>a) no history of disease (end point)*<br>b) no description of source	They were clinically underwent the Phototest, a brief cognitive test designed to screen for cognitive impairment/dementia in the primary healthcare setting.	a*
**Comparability**<br>5) Comparability of cases and controls on the basis of the design or analysis<br>a) study controls for age *	Aged 50 or older.	a*
b) study controls for any additional factor *	The presence of depressive illness was excluded.	b*

**Exposure**<br>6) Ascertainment of exposure<br>a) secure record (eg surgical records) *<br>b) structured interview where blind to case/control status*<br>c) interview not blinded to case/control status<br>d) written self-report or medical record only<br>e) no description	The degree of periodontitis was defined by the percentage of sites with loss of attachment > 3 mm as follows: 0% = absent; 0-32% = mild; 33-66% = moderate; and 67-100% = severe.	a*
7) Same method of ascertainment for cases and controls<br>a) yes*<br>b) no	As stated above.	a*
8) Non-Response rate<br>a) same rate for both groups *<br>b) non respondents described<br>c) rate different and no designation secure record (eg surgical records)		b
Risk of bias		6/9(Moderate)
Finally quality assessment		Moderate
* point		

1.9 Holmer J, Eriksdotter M, Schultzberg M, Pussinen PJ, Buhlin K (2018) Association between periodontitis and risk of Alzheimer's disease, mild cognitive impairment and subjective cognitive decline: A case-control study.

Case-control study	Holmer J-2018	
**Selection**<br>1) Is the case definition adequate<br>a) yes, with independent validation*<br>b) yes, eg record linkage or based on self-reports<br>c) no description	A medical history and a medical examination.	a*
2) Representativeness of the cases<br>a) consecutive or obviously representative series of cases*<br>b) potential for selection biases or not stated	Cases were consecutively enrolled from the Karolinska Memory Clinic at the Karolinska University Hospital in Huddinge, Sweden.	b
3) Selection of Controls<br>a) community controls*<br>b) hospital controls<br>c) no description	Controls were enrolled continuously during the study period by a random sample from The Swedish Population Register.	a*
4) Definition of Controls<br>a) no history of disease (end point)*<br>b) no description of source	Cognitive screening and medical history.	a*
**Comparability**<br>5) Comparability of cases and controls on the basis of the design or analysis<br>a) study controls for age *<br>b) study controls for any additional factor *	The controls were matched by age and gender with the combined cases group.	a*
	All patient with depression and/or signs of ongoing depression were excluded	b*

**Exposure**<br>6) Ascertainment of exposure<br>a) secure record (eg surgical records) *<br>b) structured interview where blind to case/control status*<br>c) interview not blinded to case/control status<br>d) written self-report or medical record only<br>e) no description	All clinical oral examinations were conducted by a single general dental practitioner (JH) according to a standardized protocol and in the same setting.	a*
7) Same method of ascertainment for cases and controls<br>a) yes*<br>b) no	As stated above.	a*
8) Non-Response rate<br>a) same rate for both groups *<br>b) non respondents described<br>c) rate different and no designation secure record (eg surgical records)	Fifty-four patients declined to participate, and 28 patients (12%) failed to respond in case group. 42 individuals (24%) could not be reached in control group.	c
Risk of bias		7/9(Low)
Finally quality assessment		High
* point		

1.10 Lee KH, Wu B, Plassman BL (2013) Cognitive function and oral health-related quality of life in older adults.

**Cross-sectional study**	Lee KH-2013	
**Selection**<br>1) Is the case definition adequate<br>a) yes, with independent validation*<br>b) yes, eg record linkage or based on self-reports<br>c) no description	A psychometrician administered a battery of neuropsychological measures that assessed verbal and visual memory, language, executive function, orientation, praxis, and reading ability according to DSM-IV.	a*
2) Representativeness of the cases<br>a) consecutive or obviously representative series of cases*<br>b) potential for selection biases or not stated	Participants were aged 70 and older, resident of West Virginia, community living.	a*
3) Selection of Controls<br>a) community controls*<br>b) hospital controls<br>c) no description	As stated above.	a*
4) Definition of Controls<br>a) no history of disease (end point)*<br>b) no description of source	A psychometrician administered a battery of neuropsychological measures that assessed verbal and visual memory, language, executive function, orientation, praxis, and reading ability.	a*
**Comparability**<br>5) Comparability of cases and controls on the basis of the design or analysis<br>a) study controls for age *<br>b) study controls for any additional factor *	Participants were aged 70 and older.	a*

**Exposure**<br>6) Ascertainment of exposure<br>a) secure record (eg surgical records) *<br>b) structured interview where blind to case/control status*<br>c) interview not blinded to case/control status<br>d) written self-report or medical record only<br>e) no description	Clinical dental status included the continuous variables number of decayed coronal and root surfaces, number of missing teeth, plaque index, and mean pocket depth.	a*
7) Same method of ascertainment for cases and controls<br>a) yes*<br>b) no	As stated above.	a*
8) Non-Response rate<br>a) same rate for both groups *<br>b) non respondents described<br>c) rate different and no designation secure record (eg surgical records)		b
Risk of bias		7/9(Low)
Finally quality assessment		High
* point		

1.11 Lee KH, Choi YY (2019) Association between oral health and dementia in the elderly: a population-based study in Korea

**Cross-sectional study**	Lee KH-2019	
**Selection**<br>1) Is the case definition adequate<br>a) yes, with independent validation*<br>b) yes, eg record linkage or based on self-reports<br>c) no description	Subjects who had a history of treatment for dementia in their medical records for 2017 were defined as patients with dementia.	a*
2) Representativeness of the cases<br>a) consecutive or obviously representative series of cases*<br>b) potential for selection biases or not stated	NHIS database	a*
3) Selection of Controls<br>a) community controls*<br>b) hospital controls<br>c) no description	As stated above.	a*
4) Definition of Controls<br>a) no history of disease (end point)*<br>b) no description of source	Those with no record of dementia treatment between 2002 (the year medical records started) and 2017 were classified as normal subjects.	a*
**Comparability**<br>5) Comparability of cases and controls on the basis of the design or analysis<br>a) study controls for age *<br>b) study controls for any additional factor *	Patients aged ≥65 years.	a*
	There was no significant difference between the two groups in depression.	b*

**Exposure**<br>6) Ascertainment of exposure<br>a) secure record (eg surgical records) *<br>b) structured interview where blind to case/control status*<br>c) interview not blinded to case/control status<br>d) written self-report or medical record only<br>e) no description	Patients who had had periodontal treatment with a diagnostic code for periodontitis (K05.2; acute periodontitis, K05.3; chronic periodontitis, K05.4; other periodontal disease, K05.6; periodontal disease unspecified) were defined as patients with periodontitis.	a*
7) Same method of ascertainment for cases and controls<br>a) yes*<br>b) no	As stated above.	a*
8) Non-Response rate<br>a) same rate for both groups *<br>b) non respondents described<br>c) rate different and no designation secure record (eg surgical records)		b
Risk of bias		8/9(Low)
Finally quality assessment		High
* point		

1.12 Martande SS, Pradeep AR, Singh SP, Kumari M, Suke DK, Raju AP, Naik SB, Singh P, Guruprasad CN, Chatterji A (2014) Periodontal health condition in patients with Alzheimer's disease.

**Cross-sectional study**	Martande SS-2014	
**Selection**<br>1) Is the case definition adequate<br>a) yes, with independent validation*<br>b) yes, eg record linkage or based on self-reports<br>c) no description	The diagnosis of individuals with AD was done by medical/neurological evaluations, psychiatric behavioral assessments, and neuropsychological evaluation.	a*
2) Representativeness of the cases<br>a) consecutive or obviously representative series of cases*<br>b) potential for selection biases or not stated	Individuals were selected from the Department of Neurology, National Institute of Mental Health and Neurosciences (NIMHANS), Bangalore, India.	b
3) Selection of Controls<br>a) community controls*<br>b) hospital controls<br>c) no description	As stated above.	b
4) Definition of Controls<br>a) no history of disease (end point)*<br>b) no description of source	The diagnosis of ND individuals was done by medical/neurological evaluations, psychiatric behavioral assessments, and neuropsychological evaluation.	a*
**Comparability**<br>5) Comparability of cases and controls on the basis of the design or analysis<br>a) study controls for age *<br>b) study controls for any additional factor *	Individuals ranging in age from 50 to 80 years were selected.	a*
	Individuals with depression were excluded	b*

**Exposure**<br>6) Ascertainment of exposure<br>a) secure record (eg surgical records) *<br>b) structured interview where blind to case/control status*<br>c) interview not blinded to case/control status<br>d) written self-report or medical record only<br>e) no description	A full-mouth comprehensive examination of periodontal condition was done for all individuals. The periodontal parameters were recorded at 6 sites per tooth.	a*
7) Same method of ascertainment for cases and controls<br>a) yes*<br>b) no	As stated above.	a*
8) Non-Response rate<br>a) same rate for both groups *<br>b) non respondents described<br>c) rate different and no designation secure record (eg surgical records)		c
Risk of bias		6/9(Moderate)
Finally quality assessment		Moderate
* point		

1.13 Okamoto N, Morikawa M, Okamoto K, Habu N, Iwamoto J, Tomioka K, Saeki K, Yanagi M, Amano N, Kurumatani N (2010) Relationship of tooth loss to mild memory impairment and cognitive impairment: findings from the fujiwara-kyo study

**Cross-sectional study**	Okamoto N-2010	
**Selection**<br>1) Is the case definition adequate<br>a) yes, with independent validation*<br>b) yes, eg record linkage or based on self-reports<br>c) no description	The MMSE (score range, 0-30) is used as a screening test for cognitive impairment. The Recall test (score range, 0-3) is a sub-item of the MMSE that evaluates the impairment of recent memory.	a*
2) Representativeness of the cases<br>a) consecutive or obviously representative series of cases*<br>b) potential for selection biases or not stated	The subjects were volunteer men and women aged 65 years or older from Nara prefecture.	a*
3) Selection of Controls<br>a) community controls*<br>b) hospital controls<br>c) no description	As stated above.	a*
4) Definition of Controls<br>a) no history of disease (end point)*<br>b) no description of source	The MMSE (score range, 0-30) is used as a screening test for cognitive impairment.	a*
**Comparability**<br>5) Comparability of cases and controls on the basis of the design or analysis<br>a) study controls for age *<br>b) study controls for any additional factor *	Aged 65 or older.	a*

**Exposure**<br>6) Ascertainment of exposure<br>a) secure record (eg surgical records) *<br>b) structured interview where blind to case/control status*<br>c) interview not blinded to case/control status<br>d) written self-report or medical record only<br>e) no description	The Community Periodontal Index (CPI) code of the World Health Organization (WHO). The highest code level identified was regarded as the maximum CPI code for the individual.	a*
7) Same method of ascertainment for cases and controls<br>a) yes*<br>b) no	As stated above.	a*
8) Non-Response rate<br>a) same rate for both groups *<br>b) non respondents described<br>c) rate different and no designation secure record (eg surgical records)	We excluded 145 individuals who were revealed by interviews to have severe visual or hearing impairment that were likely to affect the cognitive function tests. And CPI absence with different percent.	c
Risk of bias		7/9(Low)
Finally quality assessment		High
* point		

1.14 Panzarella V, Mauceri R, Baschi R, Maniscalco L, Campisi G, Monastero R (2020) Oral Health Status in Subjects with Amnestic Mild Cognitive Impairment and Alzheimer's Disease: Data from the Zabút Aging Project.

**Case-control study**	Panzarella V-2020	
**Selection**<br>1) Is the case definition adequate<br>a) yes, with independent validation*<br>b) yes, eg record linkage or based on self-reports<br>c) no description	The diagnosis of dementia was ascertained by specialists according to the DSM-IV-TR criteria and probable AD was diagnosed according to the National Institute on Aging and the Alzheimer's sociation criteria.	a*
2) Representativeness of the cases<br>a) consecutive or obviously representative series of cases*<br>b) potential for selection biases or not stated	Participants were recruited during the 10-year follow-up of the Zabút Aging Project (ZAP), a population-based cohort study conducted in a rural community with a low educational level in the province of Agrigento (Sicily, Italy).	a*
3) Selection of Controls<br>a) community controls*<br>b) hospital controls<br>c) no description	As stated above.	a*
4) Definition of Controls<br>a) no history of disease (end point)*<br>b) no description of source	All subjects were examined in accordance with a comprehensive standardized protocol and a comprehensive neuropsychological battery were administered to all participants by psychologists in neuropsychology.	a*

**Comparability**<br>5) Comparability of cases and controls on the basis of the design or analysis<br>a) study controls for age *<br>b) study controls for any additional factor *	There was no significance between the two groups.	a*
	Depressive symptoms were also performed in all participants.	b*
**Exposure**<br>6) Ascertainment of exposure<br>a) secure record (eg surgical records) *<br>b) structured interview where blind to case/control status*<br>c) interview not blinded to case/control status<br>d) written self-report or medical record only<br>e) no description	The Community Periodontal Index (CPI) were used to assess the periodontal status of the enrolled subjects, following the recommendation of WHO for community-based screening programs.	a*
7) Same method of ascertainment for cases and controls<br>a) yes*<br>b) no	As stated above.	a*
8) Non-Response rate<br>a) same rate for both groups *<br>b) non respondents described<br>c) rate different and no designation secure record (eg surgical records)		c
Risk of bias		8/9(Low)
Finally quality assessment		High
* point		

1.15 Shin HS, Shin MS, Ahn YB, Choi BY, Nam JH, Kim HD (2016) Periodontitis Is Associated with Cognitive Impairment in Elderly Koreans: Results from the Yangpyeong Cohort Study.

**Case-control study**	Shin HS-2016	
**Selection**<br>1) Is the case definition adequate<br>a) yes, with independent validation*<br>b) yes, eg record linkage or based on self-reports<br>c) no description	MMSE-KC scores were classified as cognitively normal (0) or cognitively impaired.	a*
2) Representativeness of the cases<br>a) consecutive or obviously representative series of cases*<br>b) potential for selection biases or not stated	One hundred eighty-four residents of Yangpyeong completed the health assessment and questionnaires.	a*
3) Selection of Controls<br>a) community controls*<br>b) hospital controls<br>c) no description	As stated above.	a*
4) Definition of Controls<br>a) no history of disease (end point)*<br>b) no description of source	MMSE-KC scores were classified as cognitively normal (0) or cognitively impaired.	a*
**Comparability**<br>5) Comparability of cases and controls on the basis of the design or analysis<br>a) study controls for age *<br>b) study controls for any additional factor *	Aged 60 or older.	a*
	There was no significant difference between the two groups in depression.	b*

**Exposure**<br>6) Ascertainment of exposure<br>a) secure record (eg surgical records) *<br>b) structured interview where blind to case/control status*<br>c) interview not blinded to case/control status<br>d) written self-report or medical record only<br>e) no description	Dental digital panoramic radiographs were used to evaluate the history of periodontitis, which was categorized into three groups according to severity of periodontitis: normal, moderate periodontitis (⩾2 interproximal sites with RABL ⩾4 mm) and severe periodontitis (⩾2 interproximal sites with RABL ⩾6 mm).	a*
7) Same method of ascertainment for cases and controls<br>a) yes*<br>b) no	As stated above.	a*
8) Non-Response rate<br>a) same rate for both groups *<br>b) non respondents described<br>c) rate different and no designation secure record (eg surgical records)		b
Risk of bias		8/9(Low)
Finally quality assessment		High
* point		

1.16 Tiisanoja A, Syrjälä AM, Tertsonen M, Komulainen K, Pesonen P, Knuuttila M, Hartikainen S, Ylöstalo P (2019) Oral diseases and inflammatory burden and Alzheimer's disease among subjects aged 75 years or older.

**Cross-sectional study**	Tiisanoja A-2019	
**Selection**<br>1) Is the case definition adequate<br>a) yes, with independent validation*<br>b) yes, eg record linkage or based on self-reports<br>c) no description	Dementia diagnoses were based on a comprehensive clinical examination carried out by a geriatrician and the diagnostic procedures according to the DSM-IV criteria.	a*
2) Representativeness of the cases<br>a) consecutive or obviously representative series of cases*<br>b) potential for selection biases or not stated	Individuals were sampled from the total population living in Kuopio, in eastern Finland.	a*
3) Selection of Controls<br>a) community controls*<br>b) hospital controls<br>c) no description	As stated above.	a*
4) Definition of Controls<br>a) no history of disease (end point)*<br>b) no description of source	Participants' cognitive function was assessed by using the Mini-Mental State Examination (MMSE).	a*
**Comparability**<br>5) Comparability of cases and controls on the basis of the design or analysis<br>a) study controls for age *<br>b) study controls for any additional factor *	Aged 75 years or older	a*

**Exposure**<br>6) Ascertainment of exposure<br>a) secure record (eg surgical records) *<br>b) structured interview where blind to case/control status*<br>c) interview not blinded to case/control status<br>d) written self-report or medical record only<br>e) no description	The periodontal pockets of all teeth were probed at two sites, mesial–buccal and distal–palatinal/lingual surfaces, but only the deepest pocket depth of each tooth was registered.	a*
7) Same method of ascertainment for cases and controls<br>a) yes*<br>b) no	As stated above.	a*
8) Non-Response rate<br>a) same rate for both groups *<br>b) non respondents described<br>c) rate different and no designation secure record (eg surgical records)		b
Risk of bias		7/9(Low)
Finally quality assessment		High
* point		

1.17 Warren JJ, Chalmers JM, Levy SM, Blanco VL, Ettinger RL (1997) Oral health of persons with and without dementia attending a geriatric clinic.

**Cross-sectional study**	Warren JJ-1997	
**Selection**<br>1) Is the case definition adequate<br>a) yes, with independent validation*<br>b) yes, eg record linkage or based on self-reports<br>c) no description	The GAC provides comprehensive diagnostic and treatment services to its patients by using a team approach that integrates several different disciplines.	a*
2) Representativeness of the cases<br>a) consecutive or obviously representative series of cases*<br>b) potential for selection biases or not stated	Subjects were recruited through the Geriatric Assessment Clinic at The University of Iowa Hospitals and Clinics.	b
3) Selection of Controls<br>a) community controls*<br>b) hospital controls<br>c) no description	As stated above.	b
4) Definition of Controls<br>a) no history of disease (end point)*<br>b) no description of source	The GAC provides comprehensive diagnostic and treatment services to its patients by using a team approach that integrates several different disciplines.	a*
**Comparability**<br>5) Comparability of cases and controls on the basis of the design or analysis<br>a) study controls for age *<br>b) study controls for any additional factor *	There was no significant difference between the two groups in depression.	a*

**Exposure**<br>6) Ascertainment of exposure<br>a) secure record (eg surgical records) *<br>b) structured interview where blind to case/control status*<br>c) interview not blinded to case/control status<br>d) written self-report or medical record only<br>e) no description	Dental assessments of the subjects were conducted by one of three dentists and one of three dental hygienists trained as recorders, and included a dental screening examination, a subject questionnaire administered verbally by the recorder, and a review of the subject's hospital record.	a*
7) Same method of ascertainment for cases and controls<br>a) yes*<br>b) no	As stated above.	a*
8) Non-Response rate<br>a) same rate for both groups *<br>b) non respondents described<br>c) rate different and no designation secure record (eg surgical records)		b
Risk of bias		5/9(Moderate)
Finally quality assessment		Moderate
* point		

1.18 Zenthofer A, Schroder J, Cabrera T, Rammelsberg P, Hassel AJ (2014) Comparison of oral health among older people with and without dementia.

**Cross-sectional study**	Zenthofer A-2014	
**Selection**<br>1) Is the case definition adequate<br>a) yes, with independent validation*<br>b) yes, eg record linkage or based on self-reports<br>c) no description	A mini-mental state examination of participants was performed by three psychologists. Participants with scores from 0 to 20 were allocated to the dementia group.	a*
2) Representativeness of the cases<br>a) consecutive or obviously representative series of cases*<br>b) potential for selection biases or not stated	The study was performed in four long-term care homes for older people all of one care society in south-west Germany.	b
3) Selection of Controls<br>a) community controls*<br>b) hospital controls<br>c) no description	As stated above.	b
4) Definition of Controls<br>a) no history of disease (end point)*<br>b) no description of source	A mini-mental state examination of participants was performed by three psychologists. Participants with scores greater than 20 were allocated to the non-dementia group.	a*
**Comparability**<br>5) Comparability of cases and controls on the basis of the design or analysis<br>a) study controls for age *<br>b) study controls for any additional factor *	Ages of participants ranged from 54 to 107 years.	a*

**Exposure**<br>6) Ascertainment of exposure<br>a) secure record (eg surgical records) *<br>b) structured interview where blind to case/control status*<br>c) interview not blinded to case/control status<br>d) written self-report or medical record only<br>e) no description	The CPITN includes 5 codes indicative of periodontal status. In the test, code 0 indicates a healthy condition, codes 1 and 2 are indicative of the presence of gingivitis, and codes 3 and 4 are indicative of moderate and severe periodontitis, respectively	a*
7) Same method of ascertainment for cases and controls<br>a) yes*<br>b) no	As stated above.	a*
8) Non-Response rate<br>a) same rate for both groups *<br>b) non respondents described<br>c) rate different and no designation secure record (eg surgical records)		b
Risk of bias		5/9(Moderate)
Finally quality assessment		Moderate
* point		

1.19 Zenthofer A, Cabrera T, Rammelsberg P, Hassel AJ (2016) Improving oral health of institutionalized older people with diagnosed dementia.

**Cross-sectional study**	Zenthofer A-2016	
**Selection**<br>1) Is the case definition adequate<br>a) yes, with independent validation*<br>b) yes, eg record linkage or based on self-reports<br>c) no description	Participants were assigned to two groups on the basis of the medical dementia diagnosis extracted from the medical records in the care documentation.	a*
2) Representativeness of the cases<br>a) consecutive or obviously representative series of cases*<br>b) potential for selection biases or not stated	Participants were recruited from four long-term care homes located in Mannheim.	b
3) Selection of Controls<br>a) community controls*<br>b) hospital controls<br>c) no description	As stated above.	b
4) Definition of Controls<br>a) no history of disease (end point)*<br>b) no description of source	Participants were assigned to two groups on the basis of the medical dementia diagnosis and MMSE extracted from the medical records in the care documentation.	a*
**Comparability**<br>5) Comparability of cases and controls on the basis of the design or analysis<br>a) study controls for age *<br>b) study controls for any additional factor *	Ages of participants ranged from 54 to 107 years.	a*

**Exposure**<br>6) Ascertainment of exposure<br>a) secure record (eg surgical records) *<br>b) structured interview where blind to case/control status*<br>c) interview not blinded to case/control status<br>d) written self-report or medical record only<br>e) no description	Dental examinations were performed and included assessment of oral hygiene, denture hygiene, and periodontal health.	a*
7) Same method of ascertainment for cases and controls<br>a) yes*<br>b) no	As stated above.	a*
8) Non-Response rate<br>a) same rate for both groups *<br>b) non respondents described<br>c) rate different and no designation secure record (eg surgical records)		b
Risk of bias		5/9(Moderate)
Finally quality assessment		Moderate
* point		

1.20 Zhu A (2019) THE CORRELATION BETWEEN MILD COGNITIVE IMPAIRMENT (MCI) AND ALZHEIMER'S DISEASE (AD) AND CHRONIC PERIODONTAL DISEASE IN ELDERLY OF TIBETAN AT HIGH ALTITUDE

**Cross-sectional study**	Zhu A-2019	
**Selection**<br>1) Is the case definition adequate<br>a) yes, with independent validation*<br>b) yes, eg record linkage or based on self-reports<br>c) no description	No description.	c
2) Representativeness of the cases<br>a) consecutive or obviously representative series of cases*<br>b) potential for selection biases or not stated	160 participants of elderly (average 64.06 years) Tibetan subjects lived in Jianza County (altitude 3,287 meters) of Qinghai Province.	a*
3) Selection of Controls<br>a) community controls*<br>b) hospital controls<br>c) no description	As stated above.	a*
4) Definition of Controls<br>a) no history of disease (end point)*<br>b) no description of source	Cognitive outcomes were assessed using MoCA.	a*
**Comparability**<br>5) Comparability of cases and controls on the basis of the design or analysis<br>a) study controls for age *<br>b) study controls for any additional factor *		

**Exposure**<br>6) Ascertainment of exposure<br>a) secure record (eg surgical records) *<br>b) structured interview where blind to case/control status*<br>c) interview not blinded to case/control status<br>d) written self-report or medical record only<br>e) no description	Periodontal examination included periodontal pocket depth, clinical attachment level, bleeding index.	a*
7) Same method of ascertainment for cases and controls<br>a) yes*<br>b) no	As stated above.	a*
8) Non-Response rate<br>a) same rate for both groups *<br>b) non respondents described<br>c) rate different and no designation secure record (eg surgical records)		b
Risk of bias		5/9(Moderate)
Finally quality assessment		Moderate
* point		

Supplementary Figures

a

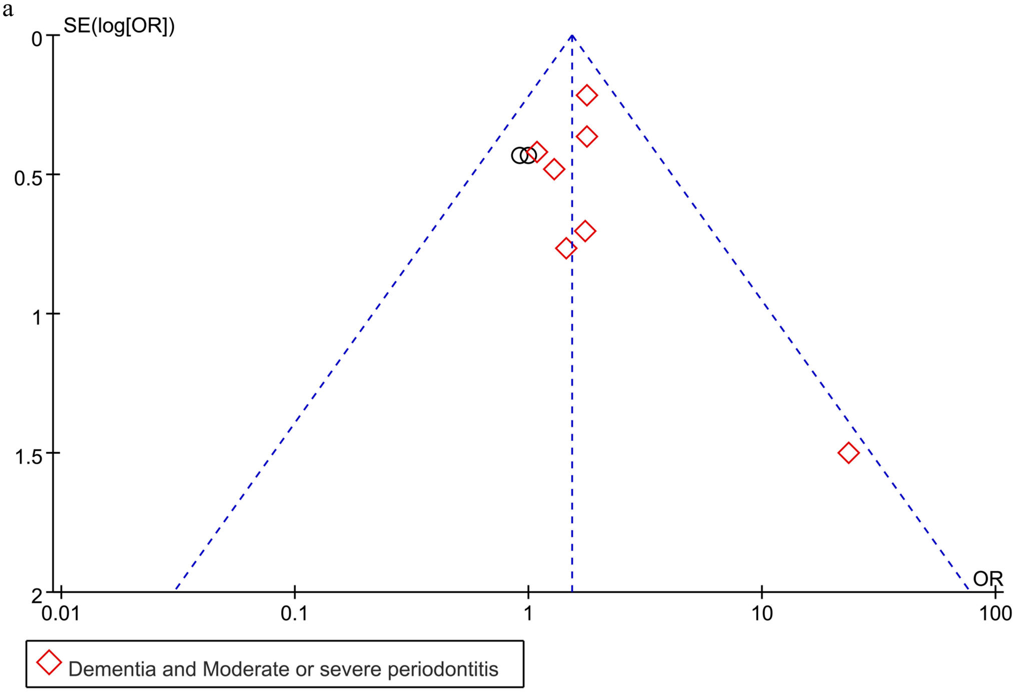

b

Study or Subgroup	Dementia Events	Dementia Total	Control Events	Control Total	Weight	Odds Ratio M-H, Random, 95% CI
Lee et al. (2019)	2950	57277	84081	1045201	0.0%	0.62 [0.60, 0.64]
Tiisanoja et al. (2019)	14	25	81	145	9.8%	1.01 [0.43, 2.36]
Zhu et al. (2019)	17	30	48	82	10.1%	0.93 [0.40, 2.16]
**Dementia and Moderate or severe periodontitis**						
Barbe et al. (2020)	20	25	11	15	3.2%	1.45 [0.32, 6.56]
Chu et al. (2015)	37	47	37	50	8.1%	1.30 [0.51, 3.33]
de Oliveira et al. (2020)	34	50	9	52	0.0%	10.15 [4.00, 25.79]
de Souza et al. (2014)	8	19	5	17	3.7%	1.75 [0.44, 6.97]
Gao et al. (2020)	86	99	79	92	10.5%	1.09 [0.48, 2.49]
Gil-Montoya et al. (2015)	109	159	126	229	40.0%	1.78 [1.17, 2.72]
Holmer et al. (2018)	25	52	26	76	13.8%	1.78 [0.87, 3.66]
Zenthofer et al. (2014)	33	33	18	24	0.8%	23.54 [1.25, 441.71]
**Subtotal (95% CI)**		**434**		**503**	**80.1%**	**1.65 [1.22, 2.22]**
Total events	318		302			
Heterogeneity: Tau² = 0.00; Chi² = 4.62, df = 6 (P = 0.59); I² = 0%						
Test for overall effect: Z = 3.27 (P = 0.001)						
**Total (95% CI)**		**489**		**730**	**100.0%**	**1.48 [1.13, 1.94]**
Total events	349		431			
Heterogeneity: Tau² = 0.00; Chi² = 7.08, df = 8 (P = 0.53); I² = 0%						
Test for overall effect: Z = 2.87 (P = 0.004)						
Test for subgroup differences: Chi² = 2.44, df = 1 (P = 0.12), I² = 59.0%						

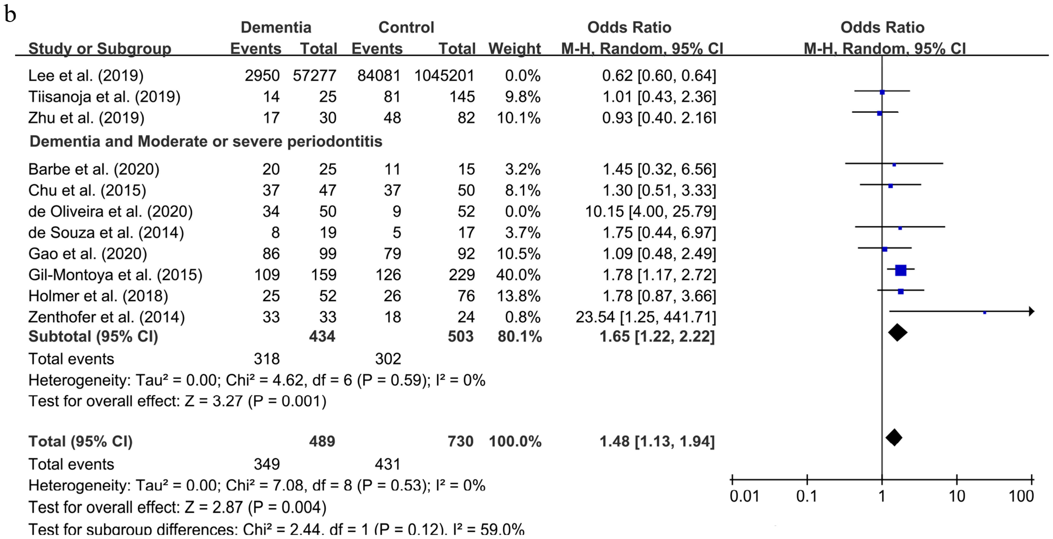

Figure S3 Funnel Plot (a) and forest plot (b) of sensitivity analysis in studies about dementia and periodontitis

a

Study or Subgroup	Dementia Mean	SD	Total	Control Mean	SD	Total	Weight	Mean Difference IV, Random, 95% CI
de Oliveira R-2020	3.4	0.2	50	2.4	0.1	52	0.0%	1.00 [0.94, 1.06]
Gil-Montoya JA-2015	3.04	0.68	159	2.6	1.5	229	34.2%	0.44 [0.22, 0.66]
Lee KH-2013	1.4	1.1	19	1.5	1.2	169	31.6%	-0.10 [-0.63, 0.43]
Martande SS-2014	4	0.75	58	2.39	0.5	60	34.2%	1.61 [1.38, 1.84]
**Total (95% CI)**			**236**			**458**	**100.0%**	**0.67 [-0.29, 1.63]**

Heterogeneity: Tau² = 0.69; Chi² = 66.82, df = 2 (P < 0.00001); I² = 97%
Test for overall effect: Z = 1.37 (P = 0.17)

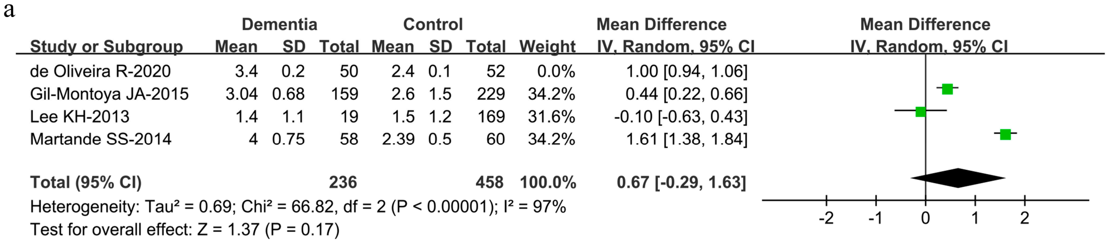

b

Study or Subgroup	Dementia Mean	SD	Total	Control Mean	SD	Total	Weight	Mean Difference IV, Random, 95% CI
de Oliveira R-2020	3.4	0.2	50	2.4	0.1	52	37.1%	1.00 [0.94, 1.06]
Gil-Montoya JA-2015	3.04	0.68	159	2.6	1.5	229	35.1%	0.44 [0.22, 0.66]
Lee KH-2013	1.4	1.1	19	1.5	1.2	169	27.8%	-0.10 [-0.63, 0.43]
Martande SS-2014	4	0.75	58	2.39	0.5	60	0.0%	1.61 [1.38, 1.84]
**Total (95% CI)**			**228**			**450**	**100.0%**	**0.50 [-0.05, 1.05]**

Heterogeneity: Tau² = 0.21; Chi² = 38.20, df = 2 (P < 0.00001); I² = 95%
Test for overall effect: Z = 1.77 (P = 0.08)

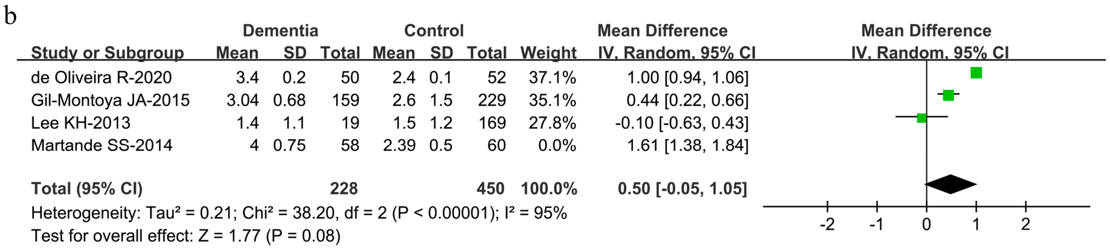

Figure S4 Sensitivity analysis in studies about periodontal status in dementia patients
